# Integrative multiomics analysis reveals association of gut microbiota and its metabolites with susceptibility to keloids

**DOI:** 10.3389/fmicb.2024.1475984

**Published:** 2024-11-28

**Authors:** Dang Li, Minghao Li, Hangqi Gao, Kailun Hu, Rongrong Xie, Jing Fan, Mingquan Huang, Chengxin Liao, Chang Han, Zhihui Guo, Xiaosong Chen, Ming Li

**Affiliations:** ^1^Nursing Department of Fujian Medical University Union Hospital, Fuzhou, China; ^2^Department of Plastic Surgery and Regenerative Medicine, Fujian Medical University Union Hospital, Fuzhou, China; ^3^Department of Plastic Surgery and Regenerative Medicine Institute, Fujian Medical University, Fuzhou, China; ^4^Engineering Research Center of Tissue and Organ Regeneration, Fujian Province University, Fuzhou, China; ^5^Department of Plastic Surgery, The Second Affiliated Hospital of Fujian Medical University, Fuzhou, China; ^6^Department of Gynecology, Fuzhou Children’s Hospital of Fujian Medical University, Fuzhou, China; ^7^Shanghai Majorbio Bio-Pharm Technology Co., Ltd., Shanghai, China

**Keywords:** keloid susceptibility, gut microbiota, metabolites, single-cell sequencing, multiomics

## Abstract

Keloid scarring is a fibroproliferative disease of the skin, which can significantly impact one’s quality of life through cosmetic concerns, physical discomfort (itchy; painful), restricted movement, and psychological distress. Owing to the poorly understood pathogenesis of keloids and their high recurrence rate, the efficacy of keloid treatment remains unsatisfactory, particularly in patients susceptible to multiple keloids. We conducted fecal metagenomic analyzes and both untargeted and targeted plasma metabolomics in patients with multiple keloids (MK, *n* = 56) and controls with normal scars (NS, *n* = 60); tissue-untargeted metabolomics (MK, *n* = 35; NS, *n* = 32), tissue-targeted metabolomics (MK, *n* = 41; NS, *n* = 36), and single-cell sequencing analyzes (GSE163973). Differences in the gut microbiota composition, plasma metabolites, and tissue metabolites were observed between the MK and NS groups; the core gut microbiota, *Oxalobacter formigenes*, *Bacteroides plebeius*, and *Parabacteroides distasonis*, were identified via the gut microbiome co-occurrence network. Single-cell data helped clarify the specific cells affected by plasma metabolites. An area under the curve analysis using a random forest model based on fecal metagenomics, plasma metabolomics, and tissue metabolomics revealed that gut bacteria, plasma, and tissue metabolites were effective in distinguishing between MK and NS groups. Decreased *Bacteroides plebeius* could lower uracil levels, altering systemic lipid metabolism, which may change the metabolic phenotype of secretory reticular fibroblasts in wounds, potentially leading to MK. These findings may open new avenues for understanding the multifactorial nature of keloid formation from the gut-skin axis and highlight the potential for novel therapeutic strategies targeting keloid lesions and the underlying systemic imbalances affected by the gut microbiome.

## Graphical abstract

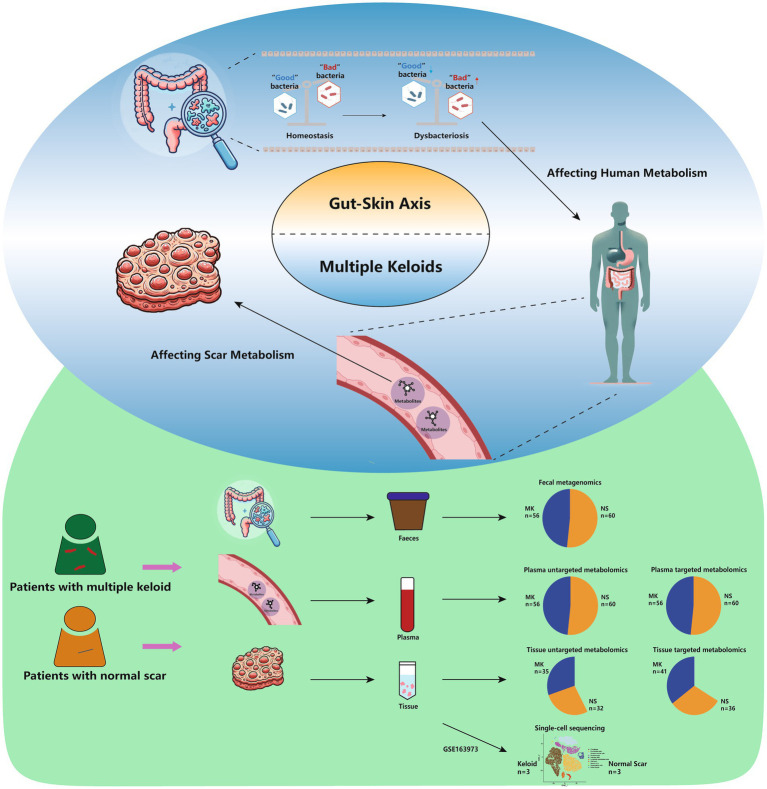

## Introduction

Keloid scarring, a fibroproliferative disease of the skin, is characterized by continued progressive expansion of the wound beyond the boundaries into the adjacent healthy skin (Limandjaja et al., 2020). In individuals prone to keloids, even minor cutaneous injuries such as insect bites, acne, and folliculitis can lead to the formation of multiple keloids (MK) throughout the body (Lee and Jang, 2018; Ogawa, 2017). Cosmetic and functional problems following keloid formation have a profound impact on patients’ quality of life and their psychological health (Chiang et al., 2016; Huang et al., 2014). Owing to the poorly understood pathogenesis of keloids and their high recurrence rate (Xu et al., 2022; Andrews et al., 2016), the efficacy of keloid treatment remains unsatisfactory, particularly in patients susceptible to MKs (Ogawa, 2022). Previous studies have predominantly focused on the pathogenesis of keloids, treating them as a uniform condition without distinguishing between single and multiple lesions. Hence, there is a pressing need for more focused studies that investigate the unique characteristics and causative factors of MK formation to better understand their development and realize more effective and personalized therapeutic approaches. Previous studies have indicated that the gut microbiota can interact with various distant organs by secreting metabolic substances (Zhao et al., 2023), and their association with many diseases in humans has been identified, such as Alzheimer’s disease, hypertension, and colon cancer (D’Argenio et al., 2022; Li et al., 2017; Zheng et al., 2020). Similarly, the gut microbiota is linked to several dermatological diseases (psoriasis, atopic dermatitis, and rosacea) via interactions with the internal body environment (Musthaq et al., 2018). Hence, many studies have introduced the concept of the gut-skin axis to uncover the connection between the gut microbiome and skin (Salem et al., 2018; Fang et al., 2022; Mahmud et al., 2022). The gut microbiota regulates skin barrier function and influences the renewal and differentiation of stem cells by secreting metabolic products such as short-chain fatty acids (SCFAs) (Salem et al., 2018). Additionally, metabolic reprogramming has been reported to play a crucial role in human conditions (Baik et al., 2019; Mossmann et al., 2023) and has been found in keloids (Li et al., 2018); therefore, examining the characteristics of the metabolites derived from gut microbiota in keloid tissues may provide a potential target for keloids.

In this study, we conducted fecal metagenomics, untargeted and targeted plasma metabolomics, untargeted and targeted tissue metabolomics, and single-cell sequencing to explore the potential correlation between the gut microbiome and MKs, which may partly explain the pathogenesis of MKs in patients. In summary, our study provides a valuable dataset and, to the best of our knowledge, is the first to describe a generalizable gut microbial and plasma/tissue signature of MK, which may offer new clues for understanding its etiology and pathogenesis.

## Materials and methods

### Participants

In this study, from June 2021 to September 2023, 116 individuals [patients with MKs (MK), *n* = 56 and controls with normal scars (NS), *n* = 60] were recruited from Fujian Medical University Union Hospital, China to undergo fecal metagenomics and untargeted/targeted plasma metabolomics; of these, 67 (MK, *n* = 35; NS, *n* = 32) were selected for tissue-untargeted metabolomics and 77 (MK, *n* = 41; NS, *n* = 36) for tissue-targeted metabolomics. The inclusion criteria were as follows: the MK group had at least three keloid lesions throughout the body, developed lesions within the past year, experienced pathological scar-related symptoms such as itching and pain, and experienced scar recurrence despite undergoing various therapies, such as resection, local radiotherapy, and drug injections. In the NS group, scar formation occurred within 2 years after injury or surgery. The exclusion criteria were as follows: (i) individuals on antibiotics, microecological preparations, immune modulators, hormonal drugs, or traditional Chinese medicine in the past month (Becattini et al., 2016); (ii) individuals with endocrine system diseases, inflammatory bowel disease, or frequent diarrhea; (iii) patients who underwent digestive system surgical procedures within the last 3 years; or (iv) patients who underwent hemodialysis, cleansing enemas, or oral bowel cleansing agents within the last 2 weeks. Written informed consent was obtained from all patients enrolled in the study. This study was approved by the Ethics Committee of the Fujian Medical University Union Hospital (No. 2021KJCX020).

### Fecal, plasma, and tissue sample collection

Fecal samples were obtained and placed in collection tubes designed for stool specimens containing a DNA stabilizer. Following this, the samples were rapidly frozen by placing them on dry ice and subsequently stored at −80°C until analysis. Blood samples were collected from the 116 participants under fasting conditions. Subsequently, these samples were centrifuged at 3,000 rpm and 4°C for 10 min, after which they were preserved at −80°C until analysis. The tissue samples were rapidly frozen on dry ice after surgery and stored at −80°C until analysis.

### Fecal DNA extraction and metagenomic sequencing

In accordance with the manufacturer’s guidelines, DNA was extracted from fecal samples using the PF Mag-Bind Stool DNA Kit (Omega Bio-Tek, United States). TBS-380 and NanoDrop2000 instruments were used to determine the concentration and purity of the extracted DNA, respectively. Additionally, DNA quality was evaluated using 1% agarose gel electrophoresis. The DNA extract was fragmented to achieve an average size of approximately 400 bp using Covaris M220 (Gene Company Limited, China). Subsequently, a paired-end library was generated using NEXTFLEX Rapid DNA-Seq (Bioo Scientific, Austin, TX, United States). Paired-end sequencing was conducted on an Illumina NovaSeq 6000 platform (Illumina Inc., San Diego, CA, United States) using the NovaSeq 6000 S4 Reagent Kit. Data were analyzed using the online platform of Majorbio Cloud Platform^1^ (Ren et al., 2022). Low-quality reads were defined as those with a length <50 bp, a quality value <20, or those containing N bases. This processing was performed using the fastp software (version 0.23.0) (Chen et al., 2018). Taxonomic and functional profiling were conducted using MetaPhlAn3 (version 3.0.14) and HUMAnN3, the next iteration of HUMAnN, the HMP Unified Metabolic Analysis Network (version 3.0.1) with default parameters (Beghini et al., 2021). Microbial species were included in our analysis if they had a minimum relative abundance of 0.01% in at least 10% of the samples; thus, 149 species were identified. Microbial pathways were annotated using the MetaCyc metabolic pathway database (Caspi et al., 2018). Linear discriminant analysis (LDA) was used to determine differential abundance and identify functional pathways in the gut microbiome.

### Untargeted and targeted metabolomic profiling of plasma/tissue samples

For untargeted metabolomic profiling, liquid chromatography-tandem mass spectrometry (LC-MS/MS) analysis was performed using Thermo UHPLC-Q Exactive HF-X system equipped with an ACQUITY HSS T3 column (100 mm × 2.1 mm i.d., 1.8 μm; Waters, United States). Pretreatment of the raw LC-MS data was conducted using Progenesis QI (Waters Corporation, Milford, MA, United States). Metabolites were identified by searching the human metabolome database,^2^ Metlin metabolomics database,^3^ and Majorbio Cloud platform (see text footnote 1). For targeted metabolomic profiling, aiming to explore short-chain fatty acid (SCFA) levels, the ExionLC AD system coupled with a QTRAP^®^ 6500+ mass spectrometer (Sciex, United States) was used for LC-MS/MS analysis of the plasma samples. Raw LC-MS data were loaded into the Sciex software OS. Automatic identification and integration of all ion fragments were performed using the default parameters, and all integrations were verified manually. Metabolite concentrations were determined using a linear regression standard curve. Data were analyzed using the online Majorbio Cloud platform (Ren et al., 2022). Metabolites were considered significantly different between the two groups if they had a variable importance in the projection (VIP) score >1 and a false discovery rate (FDR) value <0.05, as determined using the orthogonal partial least squares discriminant analysis model and Student’s *t*-test, respectively. To examine the potential functions of metabolites, metabolic features were annotated using the Kyoto Encyclopedia of Genes and Genomes (KEGG) database (Ogata et al., 1999).

### Gut microbiome co-occurrence network

To investigate the different interaction models of the gut microbiota, a co-occurrence network was constructed in the MK and NS groups using 149 differentially abundant species. The co-occurrence network included only Spearman’s correlations that met the following criteria: *p* < 0.05, |correlation coefficient| >0.3, and the top 200 correlation coefficients. Based on within-module connectivity Zi, (measuring how well a node was connected to other nodes in its module) and among-module connectivity Pi, (measuring how well a node was connected to nodes in different modules), the nodes were categorized into four groups: module hubs (Zi >2.5 and Pi <0.62), connectors (Zi <2.5 and Pi >0.62), network hubs (Zi >2.5 and Pi >0.62), and peripherals (Zi <2.5 and Pi <0.62) (Olesen et al., 2007; Guimerà and Nunes Amaral, 2005). In the co-occurrence network, the core species were evaluated using the ZiPi score (ZI <2.5, Pi >0.62).

### Single-cell RNA-sequence data processing

To further investigate the specific cell types influenced by host metabolism, we explored linoleic acid and glycerophospholipid metabolism at single-cell resolution via scMetabolism (Wu et al., 2022). Single-cell sequencing data were downloaded from The Gene Expression Omnibus,^4^ accession number GSE163973 (Deng et al., 2021). For each sample, we eliminated cells with unique molecular identifier counts of >6,000 or <200 to filter out unwanted variations and low-quality cells. Additionally, cells identified as doublets using DoubletFinder (McGinnis et al., 2019) were removed to preclude doublet-related biases. We defined the top 2,000 most variable genes based on their average expression and dispersion as highly variable genes (HVG). We reduced data dimensionality by performing the principal component analysis on the HVG. The first 30 principal components were selected for clustering. Data visualization was achieved by applying unsupervised t-distributed stochastic neighbor embedding to the cell loadings of selected principal components and utilizing cluster assignments from graph-based clustering.

### Correlation and statistical analysis

Spearman’s rank correlation coefficient was used to assess the associations between fecal metagenomics, plasma metabolomics, and tissue metabolomics. We only displayed those correlations with a *p* < 0.05 in the heatmap. The data were randomly divided into training (70%) and testing (30%) datasets for the random forest (RF) model using the randomForest package (version 4.7-1.1). Variable importance was assessed based on the mean decrease in accuracy. Receiver operating characteristic (ROC) curves and area under the curve (AUC) were calculated using SPSS (version 27.0.1.0). *p* < 0.05 was adopted for statistical significance. Additionally, the FDR *p*-value was computed for multiple comparisons using the Benjamini–Hochberg method.

## Results

### Study overview

In the present study, 116 participants were recruited from Fujian Medical University Union Hospital (Supplementary Table S1). No significant differences in age, sex, or body mass index were observed between the MK and NS groups. Metagenomic sequencing was used to generate gut metagenomic data, whereas untargeted/targeted metabolomic profiling was used to obtain plasma/tissue metabolomics data. To elucidate the potential pathogenic mechanism of MK, we compared MK and NS group patients using fecal metagenomics and plasma and tissue metabolomics. An overview of this study is shown in graphical abstract.

### Gut microbiome signatures of the MK and NS groups

**Figure 1:**
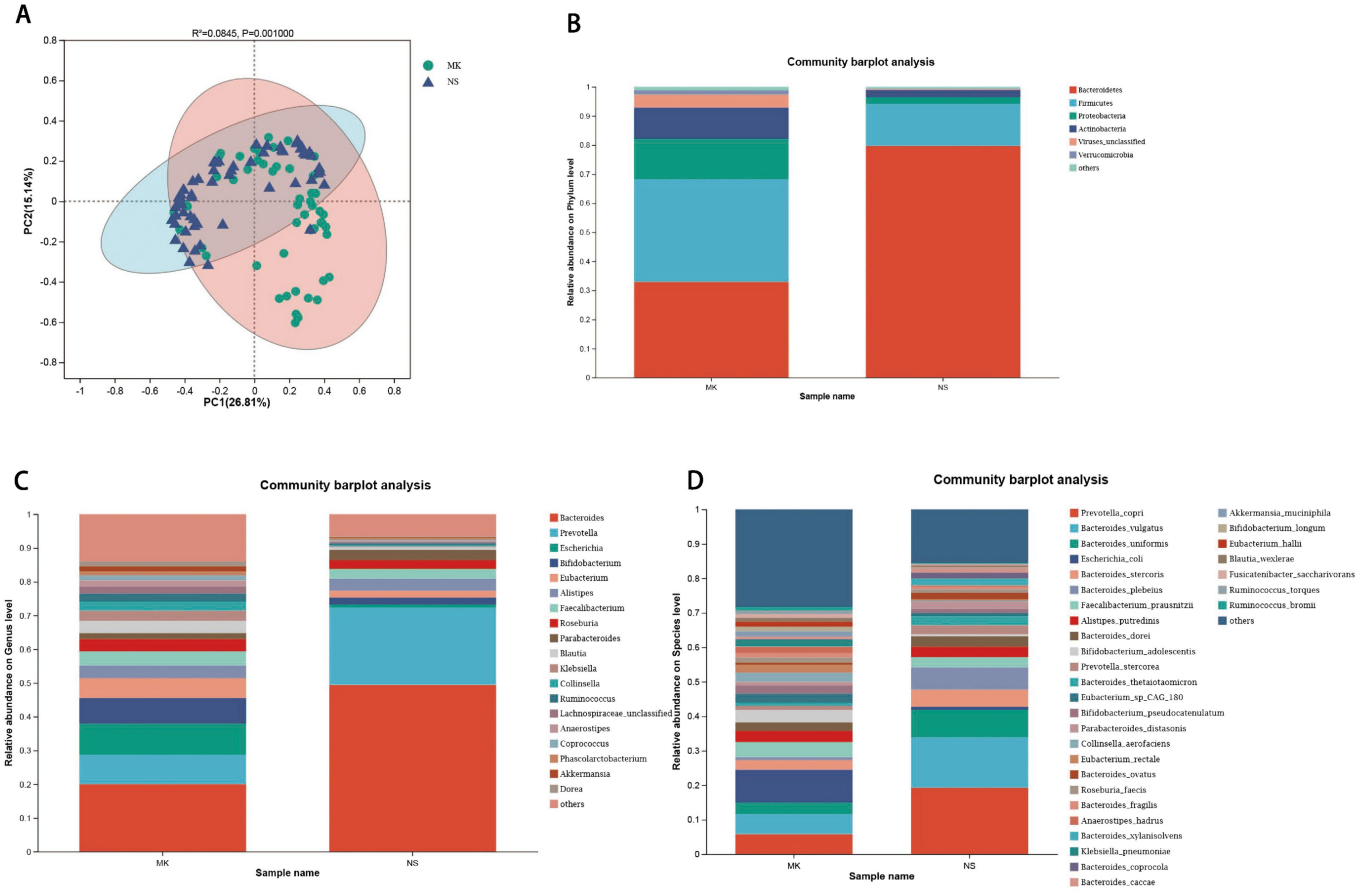
Alterations in the gut microbiome. (A) The β-diversity is shown by principal coordinate analysis based on the Bray–Curtis dissimilarity index (ANOSIM test, *p* = 0.001). Bar plots show the average relative abundance of gut microbiota in the multiple keloid (MK) and normal scar (NS) groups at the (B) phylum, (C) genus, and (D) species levels, and on the right side of the bar plots, the bacterial names are listed, with the order from top to bottom indicating a decrease from higher to lower average relative abundance.

We first analyzed the gut microbial composition of 116 fecal samples by metagenomic sequencing. We found significant differences in α-diversity between the MK and NS groups, revealed by Chao, Shannon, and Simpson indices (Supplementary Figures S1A–C). β-diversity was evaluated by PCoA analysis (*p* = 0.001, ANOSIM test) (Figure 1A) and hierarchical clustering tree (Supplementary Figures S2–S4), which indicated that the gut microbiota community of patients with MK was significantly different from that of the NS group. Next, we constructed Venn diagrams and bar plots to illustrate the differences in the overall gut microbiota composition between the MK and NS groups. Venn diagrams demonstrated that the MK and NS groups shared 9 phyla, 70 genera, and 149 species (Supplementary Figures S5B–D). The bar plots showed the relative abundances at the phylum, genus, and species levels. At the phylum level, in the NS group, *Bacteroidetes* was the most abundant phylum, followed by *Firmicutes*. However, in the MK group, the relative abundance of *Firmicutes* was higher than that of *Bacteroidetes* (Figure 1B). At the genus level, the relative abundance of *Bacteroides* in MK was distinctly different from that in NS. Moreover, the proportion of *Prevotella* was larger than that of *Bifidobacterium* in NS (Figure 1C). At the species level, the gut microbiome of NS controls presented a larger relative abundance of *Bacteroides vulgatus*, *Bacteroides uniformis*, *Bacteroides plebeius*, and *Bacteroides thetaiotaomicron* and a lower abundance of *Escherichia coli* than the gut microbiome of patients with MK (Figure 1D).

Next, linear discriminant analysis effect size (LEfSe) was used to analyze the taxonomic profiles and ascertain disparities in the gut microbiome between the MK and NS groups. A total of 49 species exhibiting differential abundance were identified (LDA >2.5, *p* < 0.05) (Supplementary Table S2). Notably, 40 of these species, including *Escherichia coli*, *Klebsiella pneumoniae*, and *Collinsella aerofaciens*, were more abundant in the MK group. Conversely, we found an abundance of nine species in NS controls, including *Bacteroides vulgatus*, *Bacteroides plebeius*, and *Bacteroides uniformis*. To elucidate the distinct microbial functions between the MK and NS groups, we analyzed the functional pathways across all metagenomes using HUMAnN3 and the MetaCyc database. The abundant pathways underwent LEfSe analysis, which revealed 54 pathways exhibiting differential abundance between MK and NS individuals (LDA >3, *p* < 0.05) (Supplementary Figure S5A). Of the 54 distinct functional pathways, 10 exhibited a notable increase in abundance in patients with MK. Our analysis revealed that sucrose biosynthesis II (PWY-7238) and glycogen degradation II (PWY-5941) pathways displayed the most significant differential increases in patients with MK and that these two pathways were predominantly influenced by *Faecalibacterium prausnitzii* (Supplementary Figures 2A,B). For NS controls, dTDP-β-L-rhamnose biosynthesis (DTDPRHAMSYN-PWY) and 6-hydroxymethyl-dihydropterin diphosphate biosynthesis I (PWY-6147) pathways were the most enriched pathways among 44 abundant functional pathways. The main contributors to these two pathways were *Prevotella copri* and *Faecalibacterium prausnitzii*, respectively (Supplementary Figures 2C,D). *Faecalibacterium prausnitzii* contributes to the gut-skin axis, which is closely associated with atopic dermatitis, a chronic, non-infectious inflammatory dermatosis (Song et al., 2016; Lee et al., 2022). Keloid scarring is a long-lasting, non-contagious inflammatory disorder. The secretory components of *Faecalibacterium prausnitzii* are also proven to modulate cutaneous wound inflammation (Stefia et al., 2020). Hence, *Faecalibacterium prausnitzii* may have a significant effect on the development of MK.

### Gut microbiome co-occurrence network in the MK and NS groups

**Figure 2:**
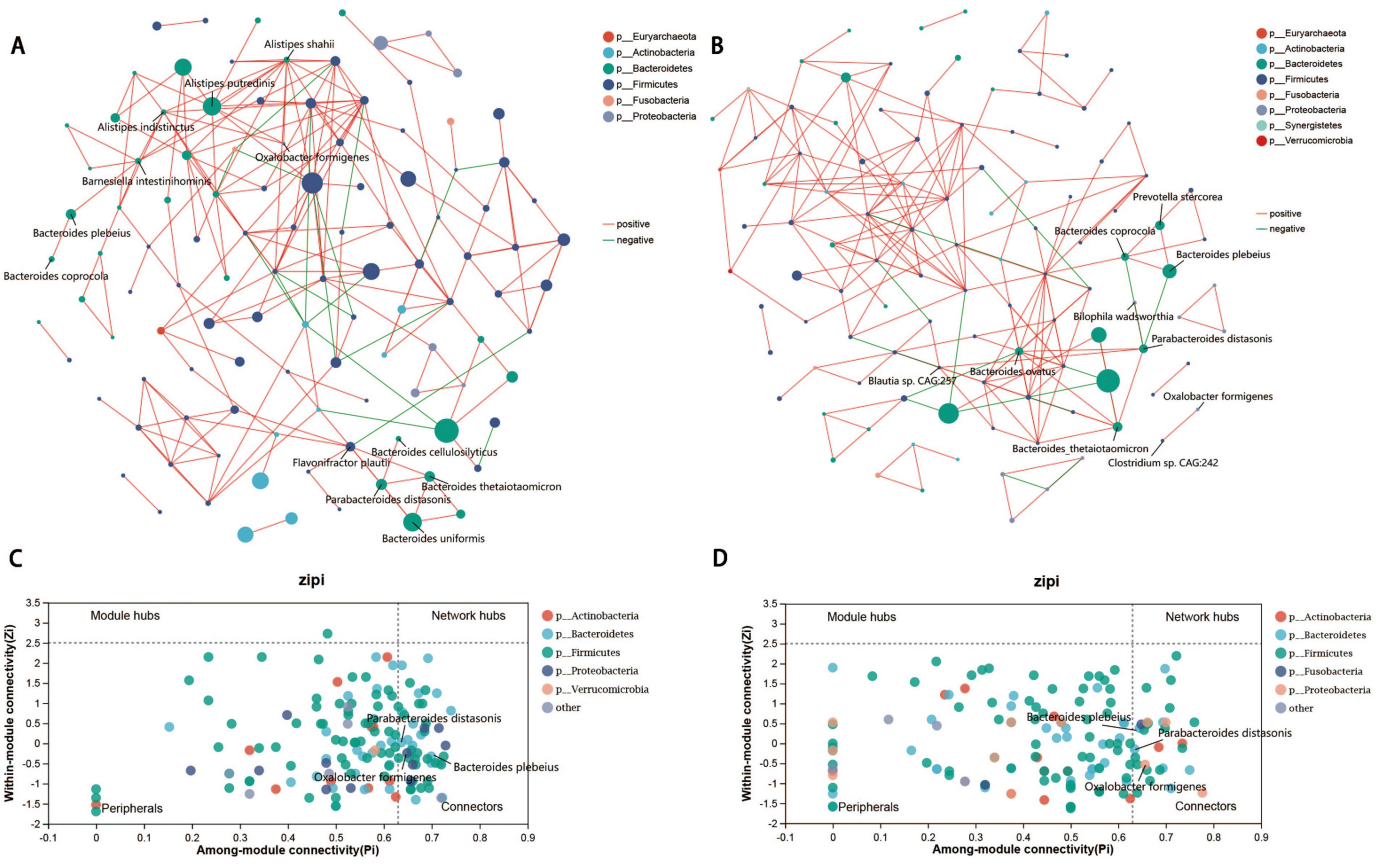
Gut microbiome co-occurrence network. Co-occurrence network of gut microbial species in patients with multiple keloid (MK, A) and normal scar (NS, B). Red lines indicate a positive correlation, whereas green lines signify a negative correlation. Zi-Pi plot illustrates the distribution of gut bacteria in (C) MK and (D) NS. The threshold values of Zi and Pi for categorizing gut bacteria are 2.5 and 0.62, respectively.

Owing to the complicated interactions within the human gut microbiome (Schmidt et al., 2018), we constructed a co-occurrence network with patients with MK and NS controls to explore the potentially different interaction models of the gut microbiota between the two groups (Figures 2A,B). Overall, the co-occurrence network of patients with MK with 149 identified species presented 182 positive and 18 negative correlations (*p* < 0.05, correlation coefficient >|0.3|, and the top 200 correlation coefficients) (Figure 2A and Supplementary Table S3). Similarly, the NS controls showed 186 positive and 14 negative correlations (Figure 2B and Supplementary Table S3). The core species in the co-occurrence network were evaluated using the ZiPi score (Supplementary Table S4) (Zi <2.5, Pi >0.62); 41 and 22 core species were identified in the co-occurrence networks of the MK and NS sites, respectively (Supplementary Table S4). *Bacteroides coprocola*, *Bacteroides plebeius*, *Parabacteroides distasonis*, *Parabacteroides merdae*, *Eubacterium rectale*, *Tyzzerella nexilis*, and *Oxalobacter formigenes* co-occurred in both MK and NS.

Subsequently, LEfSe analysis revealed that *Oxalobacter formigenes* was significantly increased in patients with MK, whereas *Bacteroides plebeius* and *Parabacteroides distasonis* were elevated in the NS controls (Supplementary Table S2). Therefore, *Oxalobacter formigenes*, *Bacteroides plebeius*, and *Parabacteroides distasonis* were deemed the most important species because they played an important role in the interaction with other microbiota within the gut microbiome, and the abundance of these three species was significantly different between the MK and NS groups (Figures 2C,D). *Oxalobacter formigenes* was positively correlated with *Alistipes putredinis*, *Alistipes shahii*, and *Alistipes indistinctus* in the MK network (Figure 2A), whereas it was positively correlated with *Clostridium* sp. CAG:242 in the NS network (Figure 2B). Moreover, *Bacteroides plebeius* was positively associated with *Barnesiella intestinihominis* and *Bacteroides coprocola* in the MK network (Figure 2A), whereas it was positively associated with both *Bacteroides coprocola* and *Prevotella stercorea* in the NS network; a negative correlation was found between *Bacteroides plebeius* and *Parabacteroides distasonis* (Figure 2B). *Parabacteroides distasonis* was positively correlated with *Bacteroides cellulosilyticus*, *Bacteroides thetaiotaomicron*, *Bacteroides uniformis*, and *Flavonifractor plautii* in patients with MK (Figure 2A), whereas it was positively associated with *Bacteroides ovatus*, *Bacteroides thetaiotaomicron*, *Bilophila wadsworthia*, and *Blautia* sp. *CAG:257* in the NS controls; a negative correlation was observed between *Parabacteroides distasonis* and both *Bacteroides coprocola* and *Bacteroides plebeius* (Figure 2B).

Similarly, psoriasis, a noninfectious chronic inflammatory condition of the skin, is characterized by a scaly erythematous eruption (Hawkes et al., 2017). A previous study revealed that following co-housing and fecal microbial transplantation trials, transplantation of gut microbiota from mice presenting an intense psoriasis-like skin phenotype aggravated psoriasiform skin inflammation in mice displaying minor symptoms. This exacerbation was accompanied by increased infiltration and differentiation of T helper 17 cells, microbiota-derived fatty acids, and abundance of *Prevotella*, but decreased levels of *Parabacteroides distasonis* (Zhao et al., 2023). Therefore, as in psoriasis, the gut microbiome may contribute to the pathogenesis of MK by modulating the host immune system, which is affected by microbiota-derived metabolites.

### Plasma metabolomics profile reveals different metabolites between the MK and NS groups

**Figure 3:**
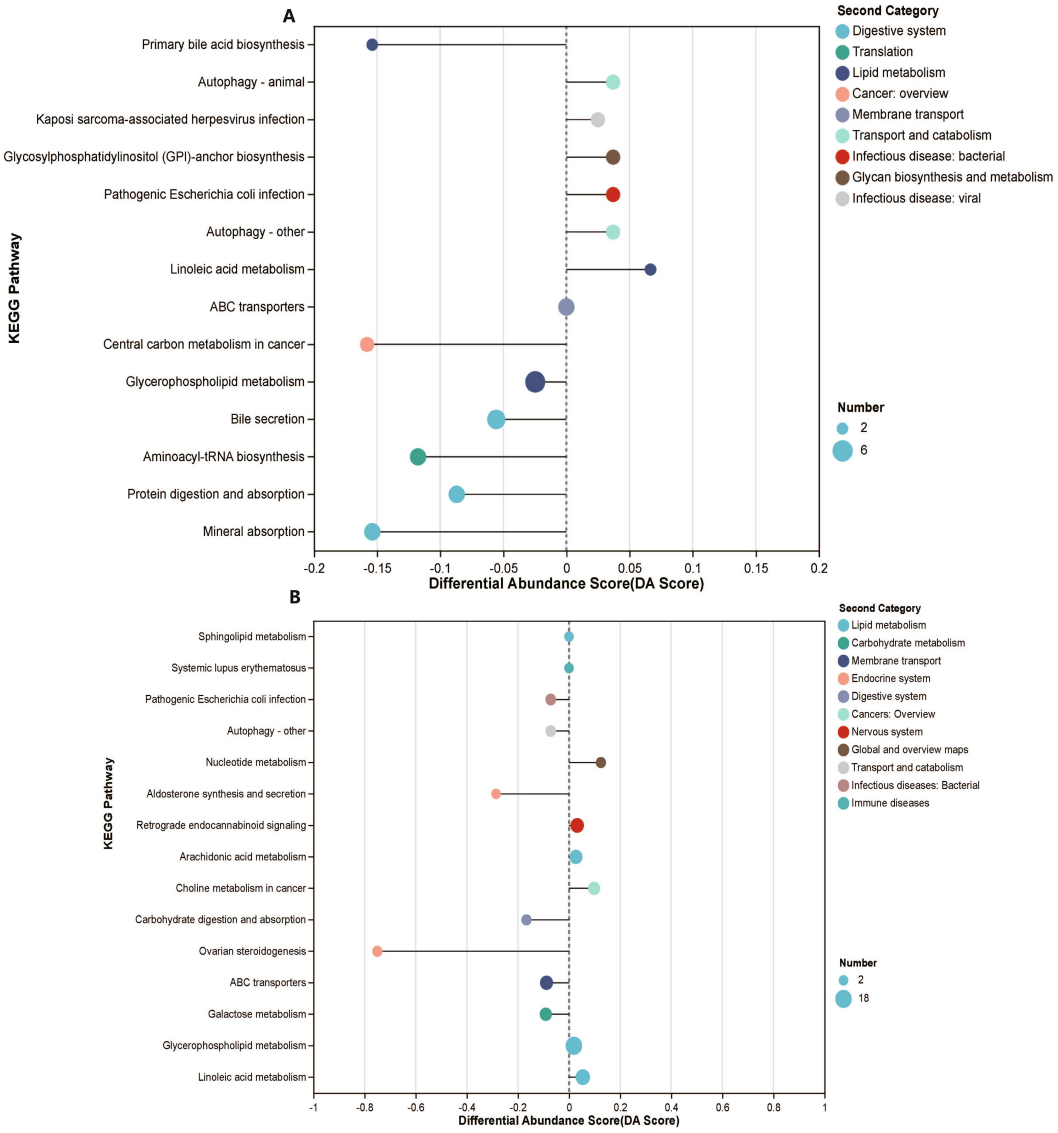
Plasma and tissue metabolomics features of participants. (A) KEGG enrichment analysis demonstrates enriched functional pathways based on 99 significantly altered metabolites. (B) KEGG enrichment analysis demonstrates enriched functional pathways based on 179 significantly altered metabolites.

To investigate the potential links between the gut microbiome and plasma metabolome, we performed untargeted metabolomics on 116 plasma samples (MK, *n* = 56, NS, *n* = 60). A total of 780 metabolites were identified, of which 99 were significantly different between the MK and NS groups (FDR <0.05, VIP >1) (Supplementary Figures S7A,B and Supplementary Table S5). In addition, 99 significantly altered metabolites were involved in 58 metabolic pathways, including fatty acid, steroid hormone, and primary bile acid biosynthesis pathways (Supplementary Table S6). Patients with MK displayed specific metabolic pathway alterations since the KEGG enrichment analysis demonstrated that 99 significantly altered metabolites were enriched in 14 pathways, including mineral absorption, bile secretion, aminoacyl-tRNA biosynthesis, protein digestion, and absorption pathways (Figure 3A). Furthermore, we observed that the primary bile acid biosynthesis and bile secretion pathways were both downregulated in patients with MK, suggesting that bile secretion was significantly lower in patients with MK than in the NS controls. Bile acids, which function as signaling molecules that regulate metabolism and inflammation via the nuclear farnesoid X receptor and Takeda G protein-coupled receptor 5 (Chávez-Talavera et al., 2017), may affect the development of local keloids by regulating metabolism and inflammation. The variation in primary bile levels between the MK and NS groups may be due to the dysbiosis of intestinal flora (Sinha et al., 2020).

Given that microbiota-derived SCFAs may be involved in a series of diseases in the host (Canfora et al., 2015; Aho et al., 2021; Fawad et al., 2022), we examined plasma SCFAs in the participants using plasma-targeted metabolomics. We quantified eight SCFAs in the plasma, of which caproic acid was significantly increased in patients with MK (FDR <0.05, VIP >1) (Supplementary Figures S3C,D and Supplementary Table S7). Similar elevated caproic acid levels have been observed in patients with multiple sclerosis than in normal controls, which is caused by immune system-mediated damage (Nakahara et al., 2012), suggesting that caproic acid exhibits pro-inflammatory characteristics attributable to the activation of the p38 MAPK signaling pathway (Saresella et al., 2020). Furthermore, in rheumatoid arthritis, an autoimmune condition characterized by chronic joint inflammation, a significant elevation in caproic acid levels was observed, but these levels markedly decreased following treatment with Danggui Sini decoction, a Traditional Chinese medicine prescription from the Treatise on Febrile Diseases (He et al., 2023). The pro-inflammatory effects of caproic acid may play a crucial role in MK pathogenesis. Targeting caproic acid-related pathways may offer novel strategies for managing MK and emphasize the importance of exploring metabolic-inflammatory links in understanding and treating MK.

### Correlation between tissue metabolomics and plasma metabolomics

**Figure 4:**
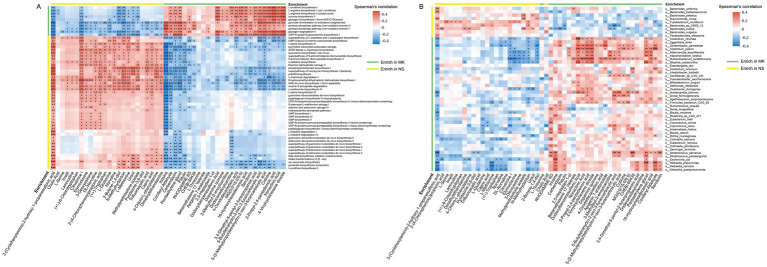
Correlation between gut microbiome and plasma metabolites. (A) The heatmap shows the associations between the enriched functional pathways of gut microbiota [multiple keloids (MK), *n* = 56, normal scars (NS), *n* = 60] and 50 differential plasma metabolites (MK, *n* = 56, NS, *n* = 60), highly associated with tissue metabolites (MK, *n* = 35, NS, *n* = 32). (B) The heatmap shows the associations between differential abundant gut bacteria (MK, *n* = 56, NS, *n* = 60) and 50 differential plasma metabolites (MK, *n* = 56, NS, *n* = 60), highly associated with tissue metabolites (MK, *n* = 35, NS, *n* = 32). ^*^p < 0.05, ^**^p < 0.01, and ^***^p < 0.001.

In our study, we did not observe significantly altered SCFAs between MK and NS via tissue-targeted metabolomics (MK, *n* = 41, NS, *n* = 36) (FDR <0.05, VIP >1) (Supplementary Figures S8A,B and Supplementary Table S8). However, 67 samples were used for tissue-untargeted metabolomics (MK, *n* = 35, NS, *n* = 32), and 882 metabolites were identified, of which 179 metabolites were significantly different between the MK and NS groups (FDR <0.05, VIP >1) (Supplementary Figures S7E,F and Supplementary Table S9). A total of 179 significantly altered metabolites were involved in 63 metabolic pathways (Supplementary Table S10) but enriched in 15 pathways between the two groups, including ovarian steroidogenesis, carbohydrate digestion and absorption, galactose metabolism, ABC transporters, and linoleic acid metabolic pathways (Figure 4B). We observed that 12-hydroxyeicosatetraenoic acid (12-HETE), 15-hydroperoxyeicosatetraenoic acid (15(S)-HpETE), and dinoprost were involved in the ovarian steroidogenesis pathway and were significantly downregulated in patients with MK. 12-HETE, 15(S)-HpETE, and dinoprost share the same arachidonic acid precursor. The deficiency of essential fatty acids, which are precursors of arachidonic acid, may be one of the causes of the formation of scar tissue (Louw, 2000a); thus, the lack of arachidonic acid may lead to a reduction in downstream metabolites that might contribute to the formation of MK.

Furthermore, we explored the correlation between tissue and plasma metabolomics (Supplementary Figure S9). Notably, 50 plasma metabolites, including 18-hydroxycorticosterone, N-methylhydantoin, and uracil, were highly correlated with tissue metabolites. Interestingly, 18-hydroxycorticosterone, an aldosterone precursor, was significantly decreased in the MK group than in the NS control in both plasma and tissue metabolomics. A previous study reported that corticosterone synthesis can be inhibited by intestinal bacteria-derived arachidonic acid (Yan et al., 2020), which suggests that reduced intestinal corticosterone synthesis might affect corticosterone levels in keloids. Overall, our data implies that plasma metabolites might affect local keloid lesions and perhaps partly explain the etiology and pathogenesis of MK.

### Correlation between gut microbiome and plasma metabolomics

Mounting evidence indicates that the pleiotropic effects of gut microbiota on host metabolism are primarily mediated by gut microbial metabolites (Canfora et al., 2019). Hence, we investigated the relationship between the 50 plasma metabolites that were highly associated with tissue metabolites and the enriched functional pathways of the gut microbiota (Figure 4A). Notably, capric acid was positively associated with the L-lysine biosynthesis III (PWY-2942) and pyrimidine deoxyribonucleoside salvage (PWY-7199) super pathways. It was negatively correlated with the glycogen degradation II (PWY-5941) and pentose phosphate pathway (non-oxidative branch) I (NONOXIPENT-PWY). Additionally, we observed elevated serotonin levels in patients with MK and found a negative correlation between serotonin and pathways such as the super pathway of L-aspartate and L-asparagine biosynthesis (ASPASN-PWY), L-lysine biosynthesis III (PWY-2942), and pyrimidine deoxyribonucleoside salvage (PWY-7199). Conversely, a positive correlation was observed between serotonin and pathways, such as UDP-N-acetyl-D-glucosamine biosynthesis I (UDPNAGSYN-PWY) and glycogen degradation II (PWY-5941). Serotonin plays a vital role in the skin, as mast cells in the skin have been reported to release serotonin (Meixiong et al., 2019), and prior research has suggested its relevance in the development of tissue fibrosis development (Sagonas and Daoussis, 2022; Dolivo et al., 2018; Dees et al., 2011). Furthermore, we found 24 significant associations between caproic acid and microbial functional pathways (Supplementary Figure S10).

Next, to identify which gut microbiota were closely associated with plasma metabolites, the correlation between 50 plasma metabolites, which were highly associated with tissue metabolites, and 49 significantly altered gut microbiota was analyzed (Figure 4B). The heatmap illustrates that each plasma metabolite exhibits at least two significant relationships with the gut microbiota. *Clostridium citroniae*, *Eggerthella lenta*, *Gordonibacter pamelaeae*, *Clostridium leptum*, *Adlercreutzia equolifaciens*, *Asaccharobacter celatus*, *Ruthenibacterium lactatiformans*, and *Bilophila wadsworthia* were significantly correlated with many plasma metabolites than the other gut microbiota. Consequently, we focused primarily on the core gut microbiota identified through the gut microbiome co-occurrence network. The heatmap revealed a positive association of *Oxalobacter formigenes* with 3-methylbut-2-enoylcarnitine, 4-vinylcyclohexene dioxide, 2-propyl-2,4-pentadienoic acid, and nonactinic acid but a negative correlation with uracil, N-methylhydantoin, 3-deoxyestrone, elymoclavine, and citrusinine I. Moreover, *Bacteroides plebeius* was positively associated with methylprednisolone acetate and uracil and negatively correlated with dodecylbenzenesulfonic acid and 5-(2-methylpropyl)tetrahydro-2-oxo-3-furancarboxylic acid. In addition, *Parabacteroides distasonis* revealed a positive relationship with 3-(cyclohexylamino)-2-hydroxy-1-propanesulfonic acid but a negative correlation with rhodamine 6G. Overall, our data demonstrated numerous significant relationships between plasma metabolites, microbial functional pathways, and microbiota, suggesting that altered microbial metabolites caused by variations in the gut microbiome might affect host plasma metabolomics in patients with MK.

### Metabolic activity at a single-cell resolution

**Figure 5:**
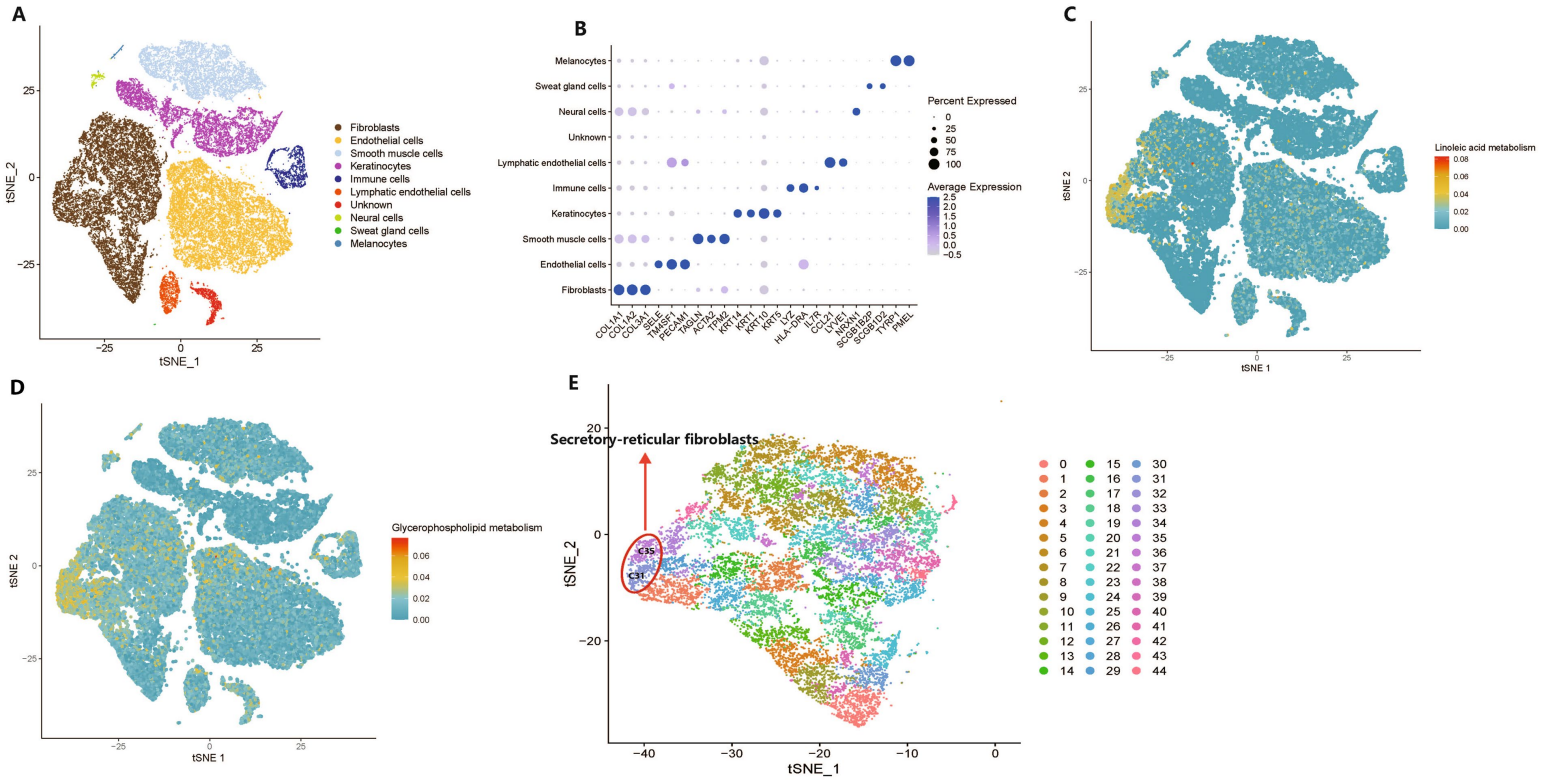
Metabolic activity at a single-cell resolution between keloids and normal scars. (A) A t-distributed stochastic neighbor embedding (t-SNE) plot shows the components in the keloids and normal scars, color-coded by cell lineages. (B) A dot plot of marker genes and their relative expression levels in all cells are shown. (C,D) The metabolic activity of linoleic acid metabolism and glycerophospholipid metabolism are shown. (E) A t-SNE plot shows the components of fibroblasts in the keloids and normal scars, color-coded by clusters.

KEGG enrichment analysis revealed 14 and 15 enriched pathways in the plasma and tissue metabolomics, respectively. Linoleic acid and glycerophospholipid metabolic pathways were both presented in the KEGG enrichment analysis of plasma and tissue metabolomics. Furthermore, the pathogenic *Escherichia coli* infection pathway was enriched in plasma and tissue metabolomics, and *Escherichia coli* was significantly increased in the MK group. These data suggest that metabolites produced by the gut microbiota may affect keloids via the circulatory system. To further investigate the specific cell types influenced by host metabolism, we explored the metabolic activity of linoleic acid and glycerophospholipid metabolism at single-cell resolution via scMetabolism (Wu et al., 2022). Unbiased clustering reveals 22 cellular clusters (Supplementary Figure S11). Based on a previous study (Deng et al., 2021), single-cell data were classified into 10 clusters (Figures 5A,B).

The scMetabolism results suggested that linoleic acid and glycerophospholipid metabolic pathways were mainly located in fibroblasts (Figures 5C,D), which play a crucial role in the development of keloids (Macarak et al., 2021); they were located in a subcluster of fibroblasts, and 45 clusters were separated from fibroblasts (Figure 5E). We found that clusters C31 and C35 had the highest metabolic activities for linoleic acid and glycerophospholipid metabolism. Recent research has indicated that normal human dermal fibroblasts comprise four distinct subpopulations: secretory-papillary, secretory-reticular, mesenchymal, and pro-inflammatory (Solé-Boldo et al., 2020). C31 and C35 were annotated as secretory reticular fibroblasts (Figure 5E). Furthermore, we found that clusters C31 and C35 were primarily derived from the NS group (Supplementary Figures S12A–E), which demonstrated that keloids may be deficient in linoleic acid and glycerophospholipid metabolism compared with normal scars. In addition, a previous study indicated that linoleic acid levels in keloids were lower than those in normal skin (Louw, 2000b). Interestingly, a study of dry eye syndrome with an inflammatory component revealed that linoleic acid may reduce ocular surface inflammation, indicating its potential anti-inflammatory properties (Barabino et al., 2003). However, a previous study highlighted the complex role of glycerophospholipids in inflammation, indicating their potential to exhibit both pro- and anti-inflammatory effects contingent on the context (Zhang et al., 2017). Taken together, the analysis of metabolic activity at a single-cell resolution revealed that metabolites from the gut microbiota may influence the tissue metabolic microenvironment, subsequently influencing the phenotype of cells, including fibroblasts.

### MK diagnosis based on microbiome and metabolomics

**Figure 6:**
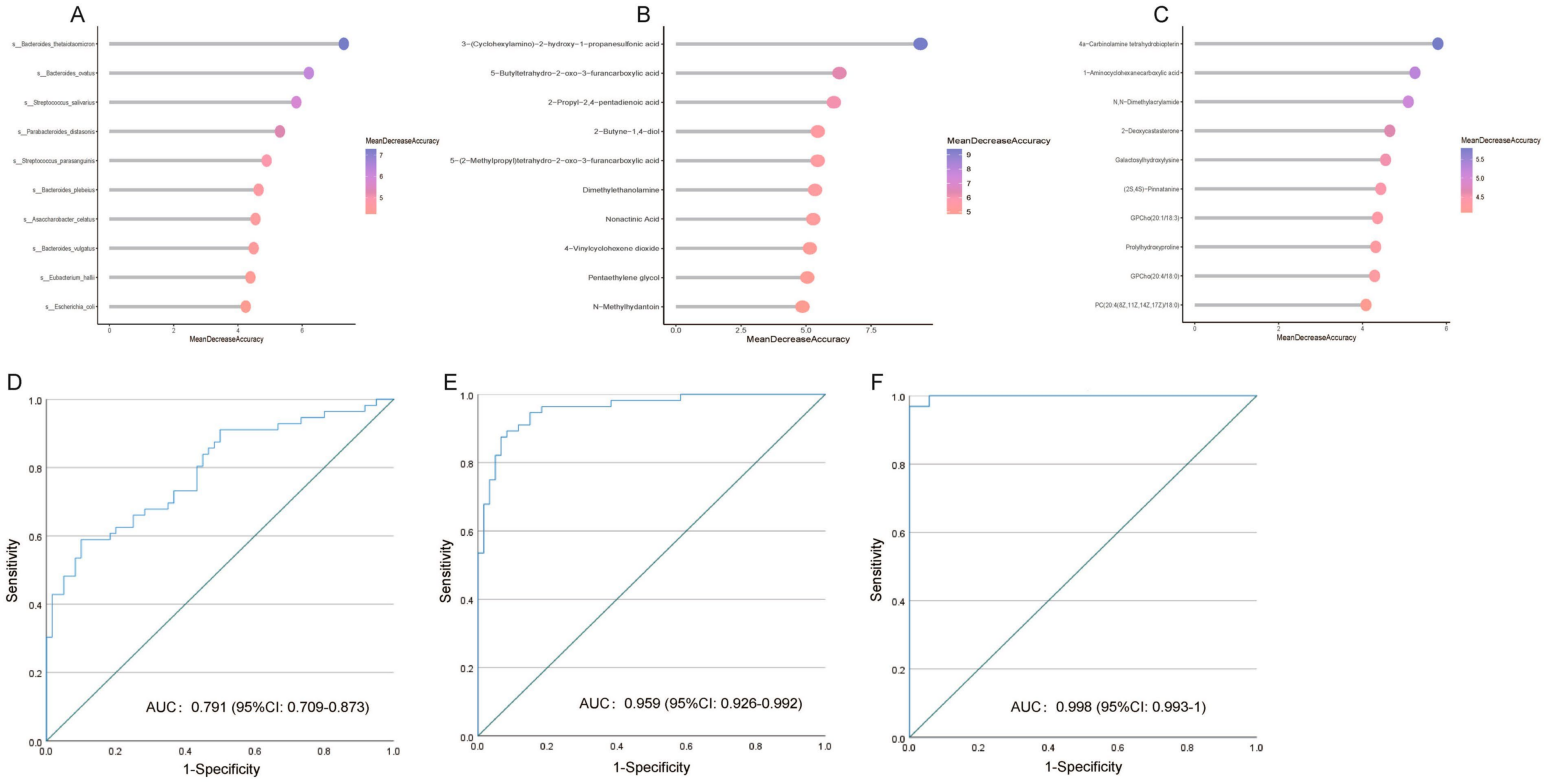
Multiple keloid (MK) prediction based on metagenomic and metabolomic features. Variable importance is indicated by the mean decrease in accuracy from random forest (RF) models based on significantly altered (A) bacteria, (B) plasma metabolites, and (C) tissue metabolites. (D) Receiver operating characteristic curve (ROC) of the RF model using gut microbiota profile to distinguish patients with MK from healthy controls. (E) ROC of the RF model using plasma metabolomic profile to distinguish patients with MK from healthy controls. (F) ROC of the RF model using tissue metabolomic profile to distinguish patients with MK from healthy controls.

Currently, MK diagnosis relies primarily on clinical manifestations. Managing MKs poses a preventive therapeutic challenge, underscoring the importance of mitigating scar formation after skin injury. Here, we investigated whether the gut microbiome, plasma metabolomics, and tissue metabolomics could serve as biomarkers for identifying high-risk patients prone to developing MKs following skin injury. Therefore, we built RF models using the relative abundances of fecal metagenomics and plasma/tissue metabolomic features. Variable importance was indicated by the mean decrease in the accuracy of the RF models. The top five gut microbiota and plasma/tissue metabolites with the highest mean decrease in accuracy scores were selected for the AUC analysis (Figures 6A–C).

Based on the fecal metagenomic data, *Bacteroides thetaiotaomicron*, *Bacteroides ovatus*, *Streptococcus sali*var*ius*, *Parabacteroides distasonis*, and *Streptococcus parasanguinis* were used as predictive markers for MK, with an AUC of 0.791 [95% confidence interval (CI): 0.709–0.873] (Figure 6D). Based on plasma metabolomics data, 3-(cyclohexylamino)-2-hydroxy-1-propanesulfonic acid, 5-butyltetrahydro-2-oxo-3-furancarboxylic acid, 2-propyl-2,4-pentadienoic acid, 2-butyne-1,4-diol, and 5-(2-methylpropyl)tetrahydro-2-oxo-3-furancarboxylic acid were used as predictive markers for MK, with an AUC of 0.959 (95% CI: 0.926–0.992) (Figure 6E). Based on the tissue metabolomics data, 4a-carbinolamine tetrahydrobiopterin, 1-aminocyclohexanecarboxylic acid, N, N-dimethylacrylamide, 2-deoxycastasterone, and galactosylhydroxylysine were used as predictive markers for MK, with an AUC of 0.998 (95% CI: 0.993–1) (Figure 6F). Our data indicate that gut bacteria and plasma and tissue metabolites are effective predictors for distinguishing patients with MK from NS controls.

## Discussion

This study based on fecal metagenomics, plasma metabolomics, and tissue metabolomics revealed that gut bacteria, plasma, and tissue metabolites were effective in distinguishing between the MK and NS groups. This study provides a valuable dataset and, to the best of our knowledge, is the first to describe a generalizable gut microbial and plasma/tissue signature of MK, which may offer new clues for understanding its etiology and pathogenesis.

The pathogenesis of keloids remains incompletely understood, and most current research primarily focuses on the pathogenesis of keloid lesions, such as the hyperactivity of fibroblasts, imbalance in collagen synthesis and degradation, and deposition of the extracellular matrix (Berman et al., 2017; Limandjaja et al., 2020). Although current research focusing on the pathogenesis of keloid lesions is comprehensive, it still fails to explain why patients with MK develop MKs on various body parts, which is a gap in understanding the susceptibility to keloids. Therefore, it is imperative to expand our investigative focus beyond localized keloid lesions to explore the systemic factors in keloid pathogenesis. A differential proportion of gut microbiota between the MK and NS groups was observed in the present study. Additionally, we found *Oxalobacter formigenes*, *Bacteroides plebeius*, and *Parabacteroides distasonis*, the core gut microbiota in the MK and NS groups, with different abundances between the two groups. Metabolomics data suggest the presence of a gut-skin axis in patients with MK, where microbiome-induced alterations in host plasma metabolomics influence keloid lesions. Subsequently, 50 plasma metabolites closely linked to the tissue metabolites were used to investigate their correlations with gut metabolomics. To the best of our knowledge, this study is the first to report an integrated analysis of the gut microbiome, plasma, and tissue metabolomics of patients with MKs.

**Figure 7:**
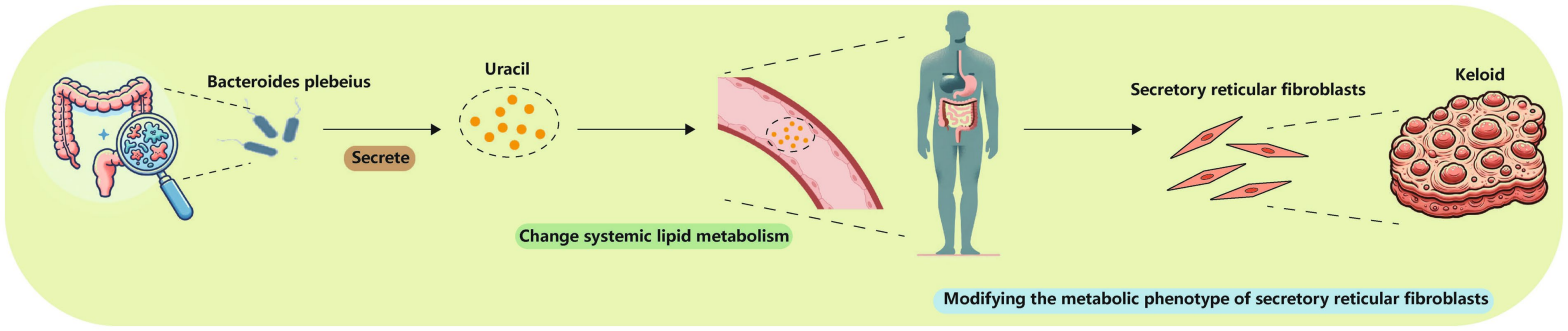
*Bacteroides plebeius* could lower uracil levels, changing systemic lipid metabolism and thereby modifying the metabolic phenotype of secretory reticular fibroblasts.

*Bacteroides plebeius* was present in a high proportion in the NS group, whereas its relative abundance was notably lower in the MK group. *Bacteroides plebeius* potentially influences the gut microbiome by enhancing probiotic abundance and mitigating damage to the intestinal mucosal barrier (Pei et al., 2022). As a probiotic, *Bacteroides plebeius* might play a role in immune system regulation, as its decreased presence has been observed in osteoarthritis, a condition characterized by low-grade inflammation (Chen et al., 2023; Molnar et al., 2021). Furthermore, a significant correlation was observed between *Bacteroides plebeius* and uracil, which is known for its role as a modulator of mucosal immunity and gut microbial homeostasis in *Drosophila* (Lee et al., 2013). Prior research has demonstrated that bacteria are capable of secreting uracil, a compound that is found to modulate lipid metabolism of its host (Whon et al., 2017). Moreover, uracil exhibits indirect anti-inflammatory effects by suppressing cyclooxygenase-2 (Kim et al., 2013; Ghoshal et al., 2011; Shao et al., 2021). Given that keloids represent a chronic, noncontagious inflammatory disorder, a decrease in *Bacteroides plebeius* could result in lower uracil levels, contributing to the disturbances in systemic metabolic regulation in MK. This was complemented by plasma and tissue metabolomics studies that showed distinct metabolic profiles in patients with MK. Subsequently, the metabolic phenotype of secretory reticular fibroblasts within wound may be altered due to systemic metabolic dysregulation, potentially resulting in MK (Figure 7).

Although the roles of the gut and skin microbiota in the host immune response differ, their synergistic effects in systemic diseases, such as scar formation, have become increasingly recognized. The gut microbiota regulates systemic immune responses through short-chain fatty acids (SCFAs) such as butyrate and propionate, while the skin microbiota primarily influences skin barrier function via local immune regulatory mechanisms (Salem et al., 2018). Certain microbes, such as *Bacteroides plebeius*, have been found to be expressed in both the gut and skin, suggesting that they may exert immunomodulatory effects through the gut-skin axis (Mahmud et al., 2022). Abundant evidence has demonstrated the significant role of SCFAs in maintaining health and contributing to disease development; they are the primary metabolites produced by gastrointestinal bacterial fermentation (Dalile et al., 2019). Targeted tissue metabolomics did not reveal any significant differences in SCFAs between the MK and NS groups. However, plasma-targeted metabolomics data revealed that the SCFA, caproic acid, was upregulated in the MK group and was positively correlated with *Rothia mucilaginosa*, *Eubacterium halli*, *Dorea formicigenerans*, *Ruthenibacterium lactatiformans*, *Sellimonas intestinalis*, and *Collinsella stercoris* but negatively associated with *Bacteroides thetaiotaomicron*. Previous research has shown that SCFAs exert pro-inflammatory effects through G protein-coupled receptors (Kim et al., 2013). Thus, gut microbiota-derived caproic acid may contribute to MK formation by regulating the inflammatory response. Glucose transporter type 4 (GLUT4) is a rate-limiting protein that facilitates glucose entry into cells and is primarily found in skeletal muscle cells. Short-chain fatty acids upregulate GLUT4 expression and facilitate its translocation to the cell membrane, thereby enhancing glucose uptake by muscle cells (He et al., 2020). However, inhibition of glycolysis leads to dose- and time-dependent suppression of keloid fibroblast proliferation via metabolic reprogramming (Li et al., 2018). Similarly, single-cell data demonstrated that glycolysis and gluconeogenesis activities were notably higher in keloid scars than in normal scars (Supplementary Figure S13). Overall, these findings suggest that SCFAs play a complex and significant regulatory role in MK and NS conditions through inflammation modulation, metabolic regulation, and cell proliferation. A deeper understanding of these mechanisms may support the development of novel therapeutic approaches and preventive strategies in patients with MK.

We developed machine learning prediction models using the gut microbiome to classify patients with and without MK. The RF classifiers demonstrated high accuracy in predicting MK and NS using the gut microbiome. Clinically, fecal microbiomes show promise as noninvasive biomarkers for distinguishing patients with MK from NS controls. Once patients were classified into the MK group, we immediately implemented appropriate scar prevention measures (Lee and Jang, 2018) to hinder or mitigate scar formation, alleviate patient discomfort, and enhance their quality of life. Additionally, using machine learning algorithms and gut microbiome network analysis, we discovered specific gut bacteria, such as *Bacteroides thetaiotaomicron* and *Bacteroides ovatus*, which exhibited a high mean decrease in accuracy scores in distinguishing the MK and NS groups, while simultaneously playing crucial roles in the overall gut microbiome. Therefore, these gut bacteria may affect the development and progression of scarring in patients with MK. This insight could lead to the development of new therapeutic strategies for manipulating the gut microbiome. By targeting these specific bacterial strains, interventions may be aimed at mitigating scar development in these patients. Managing patients with MK poses a significant challenge, as these individuals often exhibit extensive keloidal scarring on various body parts, with MKs frequently coalescing. This complicates surgical excision, which typically requires multiple surgeries complemented by adjuvant radiotherapy. Despite these interventions, the recurrence rate of keloid lesions is notably high. This high rate of keloid recurrence after treatment highlights a significant gap in current therapeutic measures and underscores the need for novel and more effective treatment strategies. Innovation in therapeutic approaches is urgently needed to prevent recurrence and minimize the need for invasive skin procedures. Advances in the understanding of the gut-skin axis can potentially revolutionize the management of keloids, ultimately translating into a marked improvement in the quality of life of those burdened by MKs.

This study has limitations. Firstly, gut microbiota exhibits regional and ethnic variations, but our study only includes Chinese subjects. Hence, further validation in diverse populations is necessary. Secondly, the lack of evidence supporting the causal relationship between gut microbiome and MK development is a common limitation in observational studies. Thus, the causal connection needs to be further verified through *in vitro* and *in vivo* models. However, keloid, especially MK, lacks recognized animal models, making it challenging to validate causal relationships between gut microbiome and MK development *in vivo*. Notably, our study may offer new insights into constructing animal models for keloid by altering the gut microbiota composition through fecal microbiota transplantation or changing the metabolic profiles of the model animals via *in vitro* injection.

## Conclusion

Our study delves into the intricate interplay between systemic factors such as fecal metagenomics, and plasma and tissue metabolomics, and the development of MKs in patients with MK. We hypothesized that an altered gut microbiota could influence systemic inflammatory responses via changing systemic metabolism, potentially triggering keloid formation on various body sites. Furthermore, we found that decreased *Bacteroides plebeius* could lower uracil levels, changing systemic lipid metabolism and thereby modifying the metabolic phenotype of secretory reticular fibroblasts in wound, which may contribute to the development of MK. These findings indicate a complex systemic network involving the gut-skin axis, where alterations in the gut microbiota and systemic metabolites may contribute to keloid pathogenesis. This systemic perspective could open new avenues for understanding the multifactorial nature of keloid formation and highlight the potential for novel therapeutic strategies targeting not only keloid lesions but also the underlying systemic imbalances.

## Data Availability

Fecal metagenomic sequencing reads is available from the Gene Expression Omnibus (GEO, http://www.ncbi.nlm.nih.gov/geo/), access number PRJNA1179461. The plasma and tissue untargeted/targeted metabolomics data are included within this article as a Supplementary material. The single-cell sequencing data is available from the Gene Expression Omnibus (GEO, http://www.ncbi.nlm.nih.gov/geo/), access number GSE163973.

## Footnotes

^1^ www.majorbio.com

^2^ http://www.hmdb.ca/

^3^ https://metlin.scripps.edu

^4^ http://www.ncbi.nlm.nih.gov/geo/
